# A Simple and Rapid Method for Standard Preparation of Gas Phase Extract of Cigarette Smoke

**DOI:** 10.1371/journal.pone.0107856

**Published:** 2014-09-17

**Authors:** Tsunehito Higashi, Yosuke Mai, Yoichi Noya, Takahiro Horinouchi, Koji Terada, Akimasa Hoshi, Prabha Nepal, Takuya Harada, Mika Horiguchi, Chizuru Hatate, Yuji Kuge, Soichi Miwa

**Affiliations:** 1 Department of Cellular Pharmacology, Graduate School of Medicine, Hokkaido University, Sapporo, Hokkaido, Japan; 2 Central Institute of Isotope Science, Hokkaido University, Sapporo, Hokkaido, Japan; University of Rochester Medical Center, United States of America

## Abstract

Cigarette smoke consists of tar and gas phase: the latter is toxicologically important because it can pass through lung alveolar epithelium to enter the circulation. Here we attempt to establish a standard method for preparation of gas phase extract of cigarette smoke (CSE). CSE was prepared by continuously sucking cigarette smoke through a Cambridge filter to remove tar, followed by bubbling it into phosphate-buffered saline (PBS). An increase in dry weight of the filter was defined as tar weight. Characteristically, concentrations of CSEs were represented as virtual tar concentrations, assuming that tar on the filter was dissolved in PBS. CSEs prepared from smaller numbers of cigarettes (original tar concentrations ≤15 mg/ml) showed similar concentration-response curves for cytotoxicity versus virtual tar concentrations, but with CSEs from larger numbers (tar ≥20 mg/ml), the curves were shifted rightward. Accordingly, the cytotoxic activity was detected in PBS of the second reservoir downstream of the first one with larger numbers of cigarettes. CSEs prepared from various cigarette brands showed comparable concentration-response curves for cytotoxicity. Two types of CSEs prepared by continuous and puff smoking protocols were similar regarding concentration-response curves for cytotoxicity, pharmacology of their cytotoxicity, and concentrations of cytotoxic compounds. These data show that concentrations of CSEs expressed by virtual tar concentrations can be a reference value to normalize their cytotoxicity, irrespective of numbers of combusted cigarettes, cigarette brands and smoking protocols, if original tar concentrations are ≤15 mg/ml.

## Introduction

Cigarette smoking is a major risk factor for cardiovascular diseases such as stroke and coronary artery disease [1], [2], for chronic pulmonary obstructive diseases [3], [4] and for several forms of cancer [5]–[7]. Cigarette smoke is reported to contain more than 4,000 chemical compounds [8], [9]. Among these are reactive oxygen species (ROS) such as peroxynitrate and free radicals of organic compounds [1], [10]. Although the free radicals are highly reactive to induce cell injury, their lifetime is too short to reach lung of smokers [10]. Recent studies indicate that cigarette smoke contain stable components which have the potential to stimulate cellular ROS production not only in the lung but also in tissues remote from the lung [11]–[13].

Cigarette smoke consists of two phases; the tar (particle) phase containing nicotine and the gas phase. In view of human health, the gas phase is important, because it can pass through the lung alveolar epithelium to reach the circulating blood and to induce damage in tissues remote from the lung [14], [15]. In fact, the gas phase extract contains stable toxic compounds which exert various cytotoxic effects in a wide range of cells [16]–[18]. In this context, we have recently shown that the gas phase extract of cigarette smoke induces cell death and plasma membrane damage through ROS generation, which is in turn induced by protein kinase C (PKC)-mediated activation of NADPH oxidase (NOX) [19], [20]. In addition, the gas phase extract of cigarette smoke can oxidize LDL in vitro, while it can promote atherosclerotic changes in aortas in vivo [14], [21], [22]. Recently, using LC/MS and GC/MS in combination with functional assays in cultured cells, we have identified several stable cytotoxic compounds responsible for cytotoxicity in the gas phase extract of cigarette smoke: among these compounds are acrolein (ACR), methyl vinyl ketone (MVK), and 2-cyclopentene-1-one (CPO) [23]. We have also shown that like the gas phase extract, these stable cytotoxic compounds induce cell damage in a PKC- and NOX-dependent manner [20], [23].

In spite of its importance for human health, no standard method for preparation of the gas phase extract of the cigarette smoke has been established, although the protocols for standard smoking protocols are established by the International Organization for Standardization (ISO) [24] and Health Canada (HC) [25]. Therefore, researchers have performed experiments using the gas phase extracts prepared by their own methods. The gas phase extracts are generally made by passing cigarette smoke through the Cambridge filter and subsequently bubbling the smoke in aqueous solution. The methods for preparation of the gas phase extracts differ mainly in terms of smoking protocols (puff smoking vs continuous smoking), bubbling conditions (pore sizes of glass ball filters for generating bubbles and temperatures of the aqueous solution) and the number of combusted cigarettes. The most serious problem is the absence of the definition for concentration of the gas phase extracts of cigarette smoke, which makes it difficult to compare the experimental data on the cigarette smoke extract from different laboratories.

In the present study, we attempt to establish a standard method for preparation of gas phase extracts of cigarette smoke, which is simple and rapid. For this purpose, we use continuous smoking protocol, because it does not require an expensive smoking machine and it is rapid. Using that smoking protocol, we optimize methods for extraction of cytotoxic activities from cigarette smoke into aqueous solution. As a measure of concentrations of the gas phase extracts of cigarette smoke, we introduce the virtual tar concentration (w/v), which is calculated on the assumption that the tar phase trapped on the Cambridge filter is dissolved in the aqueous solution used for extraction of cigarette smoke. We show that the virtual tar concentration can be used as a reference value to normalize the cytotoxic activities of gas phase extracts of cigarette smoke, irrespective of smoking conditions (continuous smoking or puff smoking), cigarette brands and the number of combusted cigarettes, as long as the original tar concentrations in the gas phase extracts are ≤15 mg/ml.

## Materials and Methods

### Materials

The cigarette used was, unless otherwise specified, the Hi-Lite (JT, Tokyo, Japan) containing 17 mg of tar and 1.4 mg of nicotine per cigarette. In some experiments, other brands such as Peace (JT, 28 mg of tar and 2.3 mg of nicotine), Seven Stars (JT, 14 mg of tar and 1.2 mg of nicotine), Mevius (JT, 10 mg of tar and 0.8 mg of nicotine), Mevius Super Light (JT, 6 mg of tar and 0.5 mg of nicotine), Marlboro (Phillip Morris, 12 mg of tar and 1.0 mg of nicotine), Lucky Strike (British American Tobacco, 11 mg of tar and 1.0 mg of nicotine), Kent 9 mg (British American Tobacco, 9 mg of tar and 0.8 mg of nicotine) were used. The materials and reagents were purchased from the following sources: Cambridge filters from Heinr Borgwaldt GmbH (Hamburg, Germany); CellTiter96 Aqueous One Solution Proliferation Assay Kit and CytoTox-One™ Homogenous Membrane Integrity Assay Kit from Promega Corporation (Madison, WI, USA); acetone, CPO and propionaldehyde from WAKO Pure Chemical Industries (Osaka, Japan); Hoechst 33342, O-(2,3,4,5,6-pentafluorobenzyl) hydroxylamine hydrochloride (PFBOA) and diphenyleneiodonium chloride (DPI) from Sigma Aldrich (St Louis, MO, USA); propidium iodide (PI) from Dojindo Laboratories (Kumamoto, Japan); ACR and MVK from Tokyo Chemical Industry (Tokyo, Japan); bisindolylmaleimide I (BIS I) from Calbiochem (San Diego, CA, USA).

### Preparation of the gas phase extract of cigarette smoke

**Figure 1:**
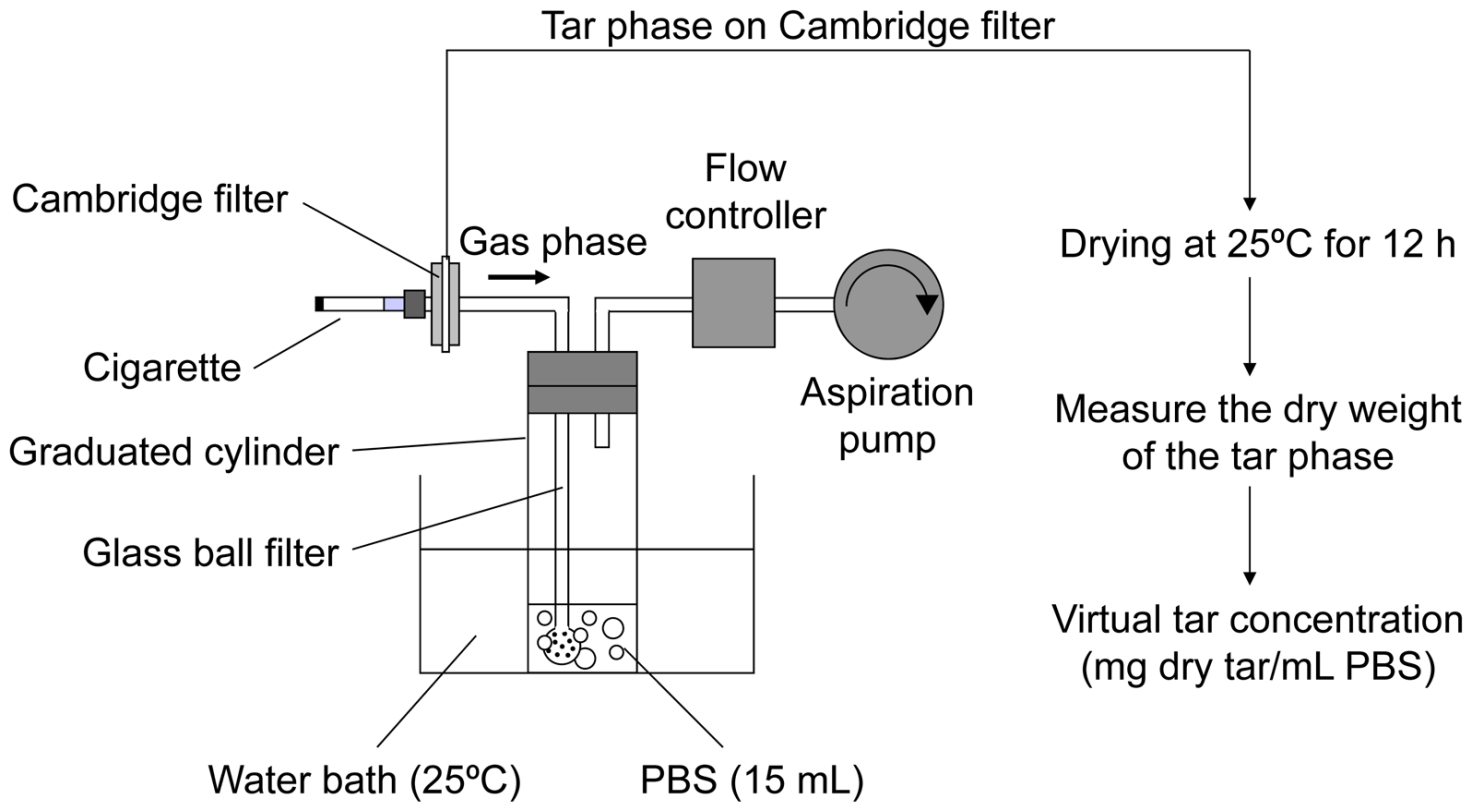
Schematic diagram of an apparatus for preparation of gas phase extracts of cigarette smoke. A standard method for preparation of the gas phase extract of cigarette smoke is as follows. Four cigarettes of Hi-Lite brand, unless otherwise specified, were sequentially combusted and the main-stream smoke was continuously sucked through a Cambridge filter at a constant flow rate of 1.050 l/min by an aspirator, to remove the tar phase. The remaining gas phase was bubbled through a glass ball filter (pore size: 20–30 µm) into phosphate buffered saline (PBS, 15 ml) in a graduated cylinder kept at 25°C. After combustion of cigarette, the filter was dried in air for 12 h at 25°C, and the dry weight of the tar phase trapped on the Cambridge filter was obtained by subtracting the weight of filter before use from that after use. The concentration of the gas phase extract was expressed as the virtual tar concentration (mg tar/ml PBS), assuming that the tar phase trapped on the Cambridge filter is dissolved in the PBS. Four cigarettes of Hi-Lite brand gave the dry tar weight of approximately 150 mg. Notably, cytotoxicity of the gas phase extracts depends not on cigarette brands but on the virtual tar concentration.

The gas phase extract of cigarette smoke (from now on, we refer to this extract as the cigarette smoke extract [CSE]) was prepared using continuous smoking protocol, unless specified otherwise: this CSE was designated cCSE. The preparation of the cCSE was performed as previously described [19], [23] with slight modifications. As shown in Fig. 1, one cigarette per trial was combusted, and the main stream of the cigarette smoke was continuously sucked through a standard glass-fiber Cambridge filter with a constant flow rate of 1.050 l/min by an aspiration pump to remove the tar phase and nicotine. According to the rules of ISO4387, cigarettes with and without filters were combusted up to 3 mm from the tipping paper and 23 mm from the end of cigarette, respectively, while the aspiration rate was set at the average flow rate of the 2-seconds puff duration of the puff smoking defined by ISO3308. The remaining gas phase of cigarette smoke was bubbled into 15 ml of phosphate buffered saline (PBS) in a 100 ml graduated cylinder (the diameter is 28 mm) at 25°C (Fig. 1). To increase the bubbling efficiency, bubbling was performed through a Kinoshita-type glass ball filter with the pore size of 20–30 µm (Kinoshita Industry, Tokyo, Japan). After combustion of cigarettes, the Cambridge filter was dried in air at 25°C for 12 h and an increase in the dry weight of the filter was regarded as the amount of the tar phase. The combustion of cigarette (usually Hi-Lite brand) was repeated, unless otherwise specified, until the dry weight of the tar phase trapped on the Cambridge filter reached 150 mg. The concentration of cCSE was expressed in terms of the virtual tar concentration which was calculated on the assumption that the tar trapped on the Cambridge filter is dissolved in the PBS used for cCSE preparation. The cCSE preparations were aliquoted and stored at −80°C until use.

For preparation of the other type of the gas phase extract of cigarette smoke by puff smoking condition (designated pCSE), smoking was performed using conditions as recommended by ISO (ISO3308). In this case, smoke was generated with a mechanical smoking machine (RM200, Heinr Borgwaldt GmbH) according to ISO3308 rules (2 s puff duration, 35 ml puff volume, bell-shaped puff profile, 60 s puff cycle). The procedures after generation of smoke were the same as those for the cCSE. To avoid the inhalation of cigarette smoke, the devices for CSE preparation were put in the fume hood.

### Cell culture

Human embryonic kidney HEK293T cells, human cervical carcinoma HeLa cells, C6 rat glioma cells, A7r5 rat aorta smooth muscle cells, EA.hy926 immortalized human umbilical vein endothelial cells (HUVEC), human lung small cell carcinoma SBC-3 cells, human lung squamous cell carcinoma H1299 cells, and human lung adenocarcinoma A549 cells were maintained in Dulbecco's modified Eagle medium (DMEM) supplemented with 10% (v/v) heat-inactivated fetal bovine serum (FBS), 100 units/ml penicillin G, and 100 µg/ml streptomycin sulfate at 37°C in humidified air with 5% CO_2_. U937 human monocytes and RAW264.7 mouse macrophages were maintained in RPMI1640 supplemented with 10% (v/v) FBS, 10 µM 2-mercaptoethanol, 100 units/ml penicillin G, and 100 µg/ml streptomycin sulfate at 37°C in humidified air with 5% CO_2_. Chinese hamster ovary (CHO) cells were maintained in F-12 Ham medium supplemented with 10% (v/v) FBS, 100 units/ml penicillin G, and 100 µg/ml streptomycin sulfate at 37°C in humidified air with 5% CO_2_.

HEK293T cells, HeLa cells, and CHO cells were purchased from RIKEN CELL BANK (Wako, Japan). C6 cells, A7r5 cells, U937 cells, RAW264.7 cells, and H1299 cells were purchased from American Type Culture Collection (Manassas, VA, USA). SBC-3 cells were purchased from Japanese Collection of Research Bioresources (Ibaraki, Japan). HUVEC were provided from Dr. Cora Jean S. Edgell, University of North Carolina at Chapel Hill [26].

### Evaluation of cytotoxicity

We used 3-(4,5-dimethylthiazol-2-yl)-5-(3-carboxymethyl)-2-(4-sulfophenyl)-2H-tetrazolium (MTS) reduction assay for evaluation of cell viability, PI uptake assay and lactate dehydrogenase (LDH) leakage assay for evaluation of cell membrane damage, and DNA fragmentation assay for evaluation of cell apoptosis.

MTS reduction assay was performed using CellTiter96 Aqueous One Solution Cell Proliferation Assay Kit, as described recently [19], [23]. Briefly, the cells were inoculated onto a 96-well plate at a density of 1×10^4^ cells per well. After incubation with CSE for 4 h, 20 µl of kit reagent was added to the culture medium (100 µl), and incubated for a further 1 h. The amount of reduced form of MTS was measured by absorbance at 490 nm using a microplate reader (SPECTRA MAX 250, Molecular Devices Corp., Sunnyvale, CA, USA). MTS reduction activity of the CSE-treated cells was represented as a percentage of the absorbance obtained from non-treated cells. MTS reduction activity in culture medium without cells was regarded as zero.

PI uptake assay was performed as recently described [19], [20], [23]. The cells were incubated with CSE for 4 h in culture medium containing 1 µg/ml PI and 1 µg/ml Hoechst 33342. The fluorescent images of PI and Hoechst 33342 were captured by IX-71 inverted fluorescent microscope (Olympus, Tokyo, Japan) equipped with ×40 objective lens (LUCPlanFL N, NA = 0.60, Olympus).

LDH leakage assay was performed using CytoTox-One™ Homogenous Membrane Integrity Assay Kit according to the manufacture's protocols as recently described [19], [20]. The cells were inoculated onto a 96-well plate at a density of 1×10^4^ cells per well. After incubation with CSE for 4 h, culture media (100 µl) were transferred to a new 96-well plate (Black Cliniplate; Thermo Fisher Scientific Inc., Rockford, IL, USA) for measurement LDH activity leaked into media. For measurement of total LDH activity, the cells cultured in parallel were disrupted by adding 2 µl of Lysis Buffer to the culture media, and the whole lysates were transferred to the 96-well plate. The enzyme reaction was started by adding 100 µl of CytoTox-ONE™ reagent. After 10-min incubation, at room temperature, the reaction was terminated by adding 50 µl of Stop Solution. The amount of the reaction product (rezorufin) was measured using a microplate spectrofluorometer (Varioscan, Thermo Fisher Scientific Inc.). LDH leakage was represented as a percentage of the total LDH activity. LDH activity in culture media without cells was regarded as zero.

For DNA fragmentation assay, the cells were inoculated onto 6-cm dish at the density of 1×10^6^ cells per dish. After 24-h incubation with CSE, the cells were lysed by incubation with lysis buffer (10 mM Tris-HCl [pH 7.4], 5 mM EDTA, 0.5% Triton X-100) for 30 min at 4°C, and centrifuged at 15,000× g for 30 min at 4°C to remove cell debris. The supernatants were transferred to new tubes, and incubated with 40 µg/ml proteinase K for 1 h at 37°C. After purification by phenol/chloroform extraction and ethanol precipitation, the DNA was reconstituted in Tris-EDTA buffer (10 mM Tris-HCl [pH 8.0], 1 mM EDTA) containing 40 µg/ml RNaseA, and incubated for 30 min at 37°C. The purified DNA was subjected to 1.8% agarose gel electrophoresis.

### Analysis of CSE by HPLC and GC/MS

Identification of cytotoxic compounds in the CSE was performed using HPLC and GC/MS as described recently [23]. Briefly, the CSE was fractionated by HPLC equipped with a reverse-phase column, each fraction was analyzed for cytotoxic activity using PI uptake assay, and two active fractions inducing PI uptake into cultured C6 glioma cells were analyzed for identification of cytotoxic compounds using GC/MS. Before analysis by GC/MS, the active fractions from HPLC were derivatized with a carbonyl reagent PFBOA to stabilize carbonyl compounds as described [23].

### Data analysis

For evaluation of cytotoxic activities of CSEs (cCSE or pCSE) using MTS reduction assay and LDH leakage assay, concentration-response curves were constructed by plotting the activities against virtual tar concentrations of CSEs. MTS reduction activity in the presence of CSEs was represented as a percentage of control value in the absence of CSEs, while LDH activity leaked into culture medium was represented as a percentage of control value in culture medium of cells lysed by 0.2% Triton X-100 in the absence of CSEs. From the concentration-response curves, the values for EC_50_ and maximum inhibition were obtained. The data for experiments performed with or without cultured cells were presented as means ± SE or means ± SD., respectively. The significance of the differences between mean values was evaluated with GraphPad PRISM™ (version 4.0, GraphPad Software Inc., San Diego, CA, USA) by student's unpaired *t*-test or one-way ANOVA, followed by Tukey's multiple comparison test. A *P* value less than 0.05 was considered to indicate statistically significant differences.

## Results

### Validation of quantification of tar amount

In the present study, we attempted to represent the concentration of the cCSE in terms of the virtual tar concentration which was calculated on the assumption that the tar phase (dry weight [mg]) trapped on the Cambridge filter is dissolved in the PBS (15 ml). Since the tar phase was reported to contain water [27], we first investigated conditions for vaporizing the water in the tar phase on Cambridge filters to estimate the dry weight of the tar phase. To minimize vaporization of volatile chemical components in the tar phase, we tested relatively low temperatures such as 25°C and 55°C for vaporization of water.

**Figure 2:**
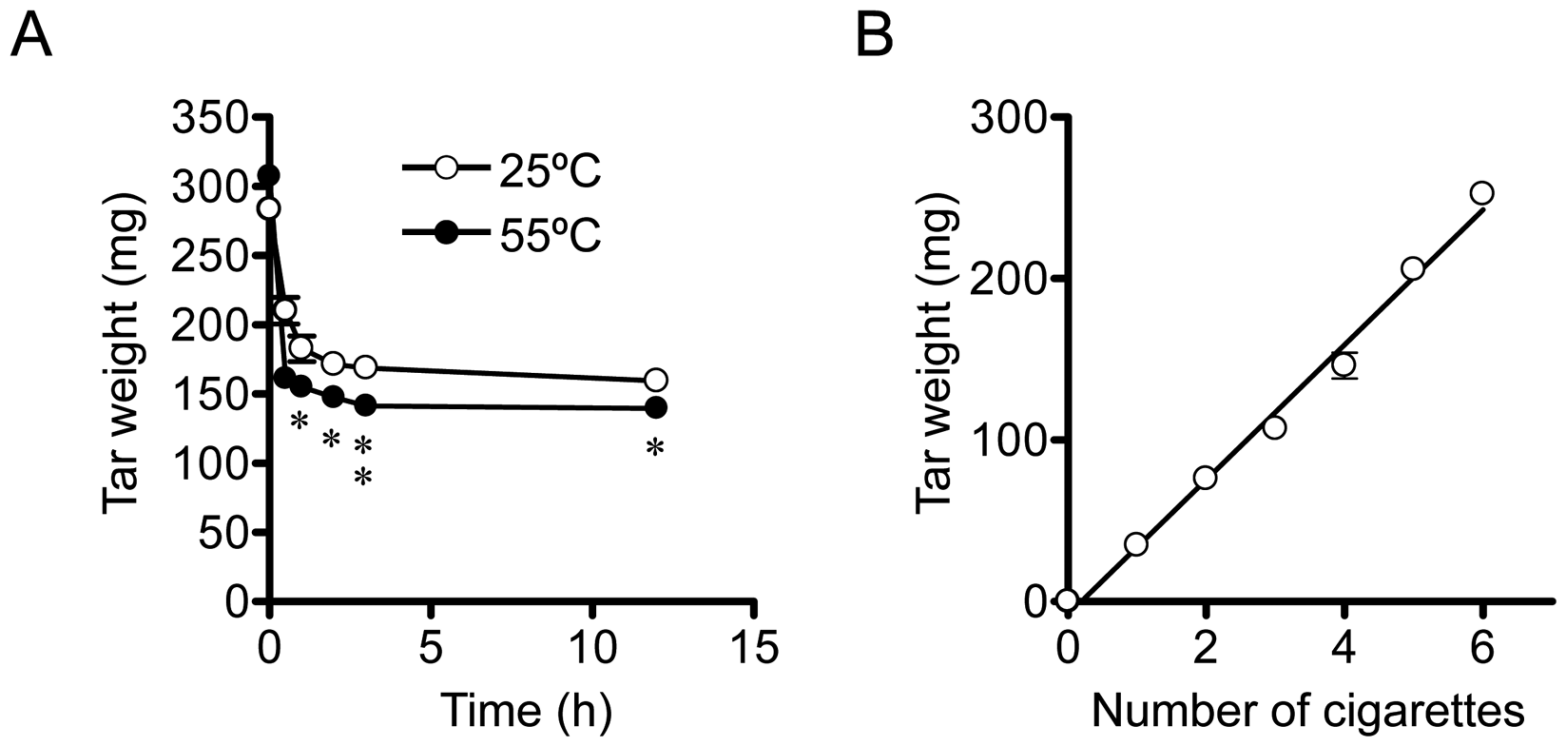
Quantification of the weight of the tar of cigarette smoke trapped on the Cambridge filter. (A) Time-course of a decrease in the weight of the tar phase of cigarette smoke trapped on the Cambridge filter after drying at 25°C (open circle) or 55°C (closed circle). Four cigarettes of Hi-Lite brand were sequentially combusted and the main-stream smoke was sucked through a Cambridge filter at a constant flow rate of 1.050 l/min by an aspiration pump. After combustion of cigarette, the filter was dried for various lengths of time at 25°C (open circle) or 55°C (closed circle), and the weight of the tar phase of cigarette smoke trapped on the Cambridge filter was obtained by subtracting the filter weight before combustion of cigarette from the weight after combustion. (B) The relationship between the number of combusted cigarettes and the dry weight of the tar phase trapped on the Cambridge filter. Various numbers of Hi-Lite brand cigarettes were sequentially combusted as described in A. After combustion, the filter was dried for 12 h at 25°C, and the dry weight of the tar phase on the Cambridge filter was determined as described in A. Values represent means ± SD of three experiments. *, P<0.05; **, P<0.01 versus 25°C.

For evaluation of vaporizing conditions, smoke from 4 cigarettes of Hi-Lite brand was passed through a Cambridge filter, and the filter was dried at 25°C or 55°C for various lengths of time, and the weight of the filter was measured. The increase in the weight of the filter following smoking was regarded as the weight of the tar phase. As shown in Fig. 2A, the weight of the tar phase on the filter decreased up to 2 h following drying at 25°C, and thereafter, it reached a plateau up to 12 h. Following drying at 55°C, the weight of the tar phase on the filter also decreased in a similar time course, and thereafter, it reached a plateau up to 12 h. Notably, the weight of the tar phase on the filter following drying at 55°C for 12 h is significantly lower than that following drying at 25°C for 12 h, indicating that part of volatile components in the tar phase might have been vaporized. Therefore, in the following experiments, Cambridge filters were dried at 25°C for 12 h.

As shown in Fig. 2B, the weight of the tar phase trapped on the Cambridge filter increased linearly with an increase in the number of combusted cigarettes up to 6 cigarettes (of Hi-Lite brand), which gave approximately 225 mg of tar on the filter. During combustion of cigarettes, the aspiration speed was continuously monitored by a Kofloc flowmeter, and it was found to be constant (1.050 l/min) up to 6 cigarettes. These results indicate that the Cambridge filter functions normally without being saturated with the tar phase at least up to 225 mg. In the following experiments, we usually used 4 cigarettes for preparation of the cCSE. When more than 4 cigarettes were combusted for cCSE preparation, a new Cambridge filter was used every 4 cigarettes to avoid saturation of the filter with tar phase.

### Effects of the temperature of PBS and the pore size of the glass ball filter on the cytotoxicity of the gas phase extract of cigarette smoke

**Figure 3:**
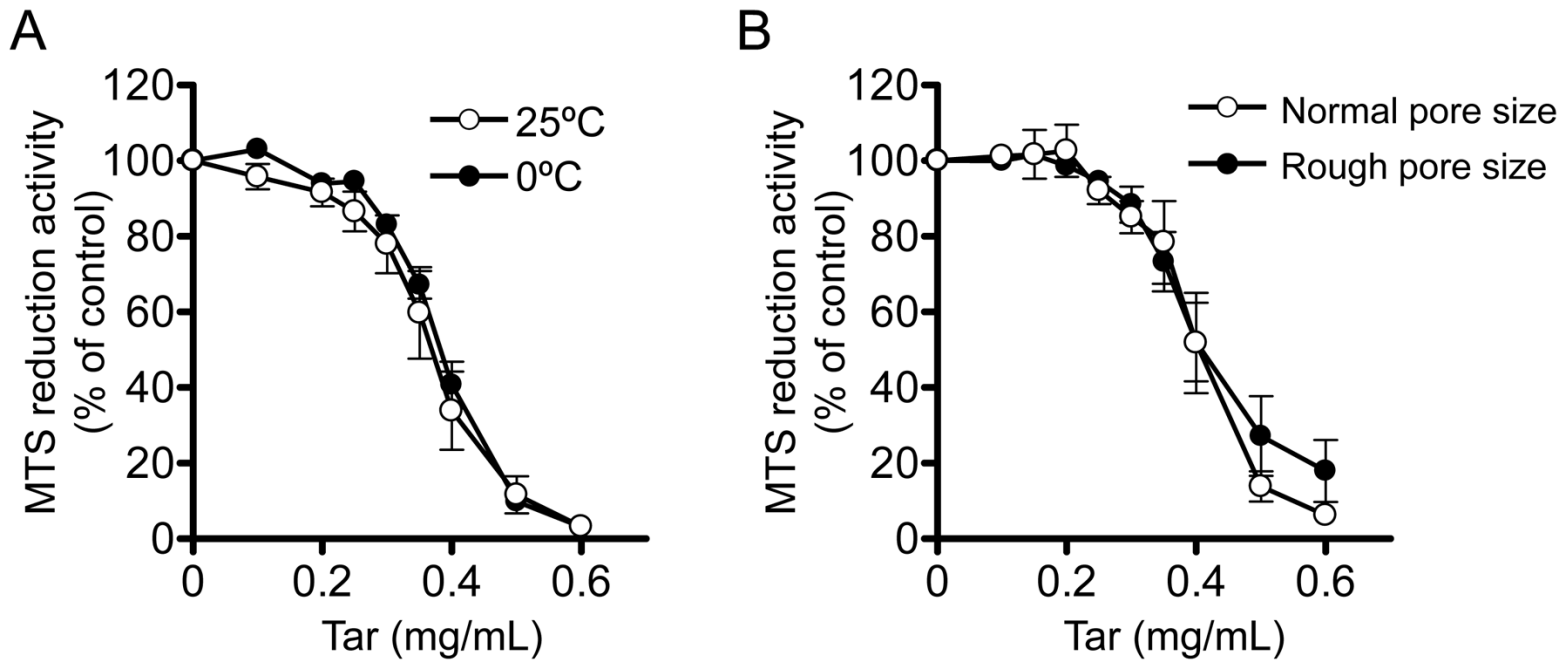
Effects of the bubbling condition on gas phase extracts of cigarette smoke. Effects of the temperature of phosphate-buffered saline (PBS) (A) and pore size of a glass ball filter for bubbling the gas phase of cigarette smoke into PBS (B) on the cytotoxic activities of the gas phase extract were examined. The gas phase extract of cigarette smoke (designated cCSE) was prepared as described in the legend for Fig. 1, by continuous smoking of four cigarettes of Hi-Lite brand which gave the virtual tar concentration of approximately 10 mg/ml PBS. In panel A, the temperature of PBS for extraction of cigarette smoke was kept at either 25°C (open circle) or 0°C (closed circle), and in panel B, the pore size of the glass ball filter was either normal (pore size, 20–30 µm; open circle) or rough (pore size, 100–120 µm; closed circle). For evaluation of the cytotoxicity of cCSE, C6 glioma cells were incubated for 4 h with various concentrations of cCSE and MTS reduction activity was determined as described in Materials and methods. MTS reduction activity in the absence of cCSE was represented as 100%. Values represent means ± SE of three experiments, each in triplicate.

Since the water-solubility of chemical compounds is generally affected by temperature, we examined the effect of the temperature of PBS in the graduated cylinder on the cytotoxicity of cCSE preparation. cCSE was prepared by continuous smoking of four cigarettes of Hi-Lite brand which gave 150 mg of tar on the Cambridge filter. The concentrations of the cCSE were expressed in terms of the virtual tar concentration, assuming that the tar trapped on the Cambridge filter was dissolved in cCSE. The tar concentration of the original cCSE was calculated to be 10 mg/ml. The cCSEs prepared with PBS (15 ml) kept at 0°C and 25°C showed similar concentration-response curves for inhibition of MTS reduction activity (Fig. 3A), indicating that the temperature of the PBS has little effect on the recovery of cytotoxic compounds.

The pore size of the glass ball filter used for bubbling smoke might affect the cytotoxicity of cCSE by changing the size of bubbles and hence the efficiency of transfer of cytotoxic compounds from bubbles to PBS. To optimize the pore size of the glass ball filter, we compared the cytotoxicity of cCSEs prepared using glass ball filters with different pore sizes. The cCSEs prepared with glass ball filters of normal pore size (20–30 µm) or large pore size (100–120 µm) showed similar concentration-response curves for inhibition of MTS reduction activity (Fig. 3B), indicating that the pore size of glass ball filters has little effect on the recovery of cytotoxic compounds. Therefore, in the following experiments, we used PBS kept at 25°C and a glass ball filter of normal pore size for preparation of the cCSE.

### Effects of the original tar concentrations of cCSEs on their cytotoxic potency

**Figure 4:**
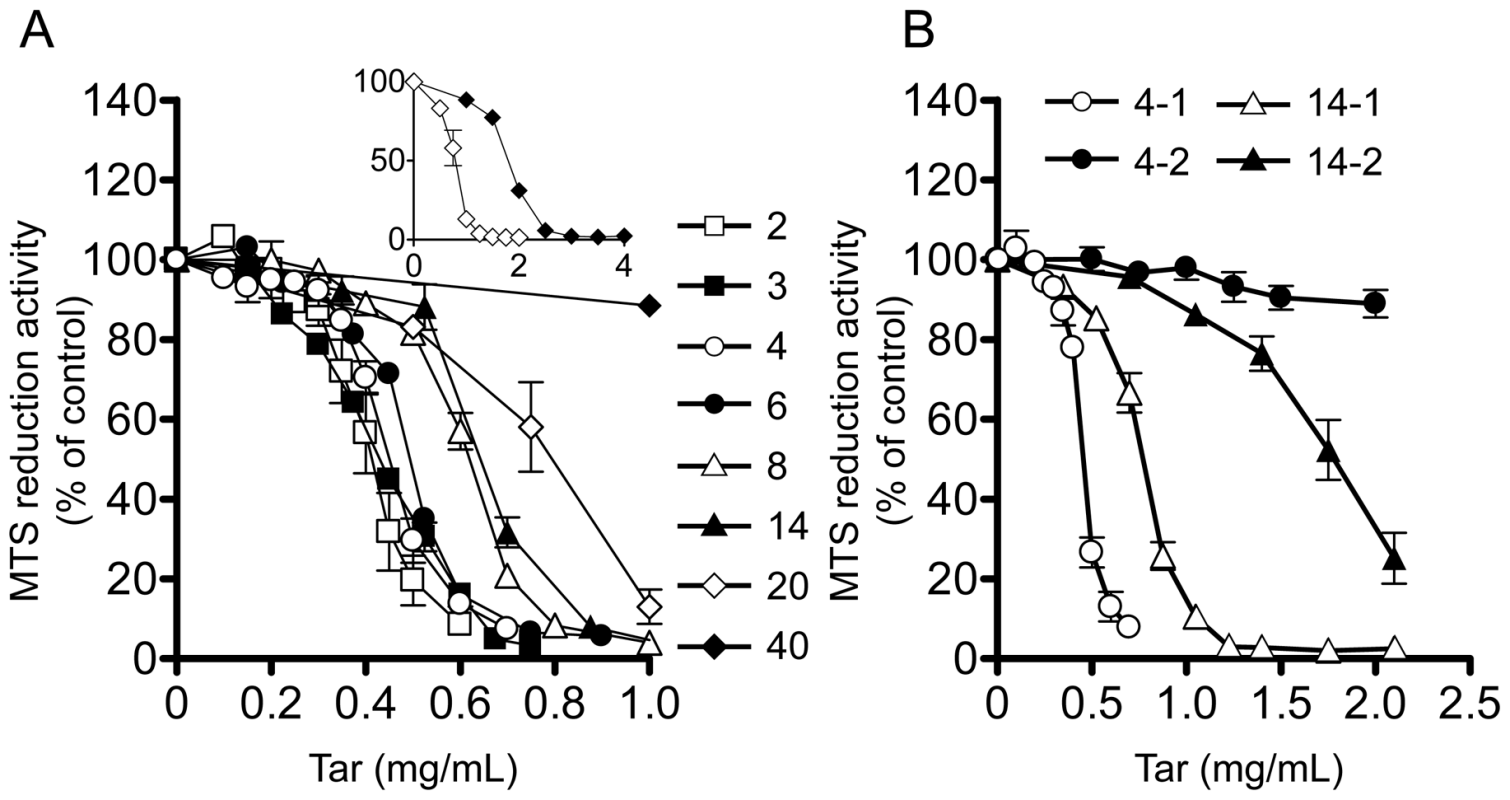
Cytotoxic activities of gas phase extracts of cigarette smoke and the number of cigarette. Concentration-response curves of the gas phase extracts of cigarette smoke prepared from varying numbers of cigarettes (Hi-Lite brand) (A) and of the phosphate buffered saline (PBS) in the second graduated cylinder (B) for inhibition of MTS reduction activity. (A) The gas phase extract of cigarette smoke (designated cCSE) was prepared from varying numbers (2–40) of cigarettes (Hi-Lite brand) based on continuous smoking protocol, while a new Cambridge filter was used every 4 cigarettes. Inset: Concentration-response curves of the cCSE prepared from 20 or 40 cigarettes with a change in scale of concentrations on x axis. (B) In the apparatus for preparation of cCSE, the second graduated cylinder with 15 ml of PBS was incorporated downstream of the first one, and cCSE was prepared from either 4 or 14 cigarettes (Hi-Lite brand). The cytotoxicity of PBS in the original and second graduated cylinders was evaluated using MTS reduction assay. MTS reduction activity in the absence of the gas phase extract was represented as 100%. Values represent means ± SE of three experiments, each in triplicate. 4-1 (14-1) and 4-2 (14-2) represent the cytotoxic activities of the PBS in the first (original) and second graduated cylinders prepared from 4 (14) cigarettes, respectively.

To examine how much cigarette smoke will saturate the PBS (15 ml), we constructed the concentration-response curves for cytotoxicity of cCSEs prepared from varying numbers of cigarettes (Fig. 4A and Table 1). In this experiment, 2–40 cigarettes of Hi-Lite brand were combusted by continuous smoking protocol, and a new Cambridge filter was used every 4 cigarettes, to avoid the saturation of the filter with tar. Again, the concentrations of the cCSE were expressed in terms of the virtual tar concentration, based on the dry weight of tar trapped on the Cambridge filter.

**Table 1:** The EC_50_ and maximal values for inhibition of MTS reduction activity of the cCSEs prepared from varying numbers (2–40) of cigarettes (Hi-Lite brand).

Number of cigarette	EC_50_ (mg/ml)^a^	Maximum inhibition (%)^a^
2	0.411±0.022	91.45±0.70
3	0.431±0.083	96.66±0.54
4	0.451±0.011	92.57±0.66
6	0.494±0.001	94.30±0.46
8	0.618±0.012**	96.95±0.61
14	0.643±0.013**	97.72±0.03
20	0.784±0.059**	97.76±0.53
40	1.797±0.024**	98.17±0.06

a The cCSEs at the original tar concentration of 10 mg/ml were subjected to MTS reduction assay in C6 glioma cells.

The concentration-response curves for inhibition of MTS reduction activity were constructed, and the EC_50_ values and maximal inhibition were determined. The concentrations of cCSEs were represented by the virtual tar concentrations. MTS reduction activity in the absence of the cCSEs was represented as 100%. Values represent means ± SE of three experiments, each in triplicate.

**P<0.01 vs the value for 2 cigarettes.

The concentration-response curves for inhibition of MTS reduction activity were similar among cCSEs which were made from 2–6 cigarettes (equivalent to the original tar concentration of 5–15 mg/ml), as demonstrated by comparable values for the EC_50_ and maximal inhibition (Fig. 4A and Table 1). However, the concentration-response curves began to be shifted to the right, when more than 8 cigarettes (equivalent to the original tar concentration ≥20 mg/ml) were used (Fig. 4A and Table 1). The rightward shift of the curves was more marked with an increase in the number of cigarettes, indicating that the cytotoxic activities of cCSEs prepared from larger numbers of cigarettes were lower than expected at a given tar concentration. These results taken together strongly demonstrate that cytotoxic activity in the smoke is efficiently extracted into PBS up to 6 cigarettes of Hi-Lite brand (equivalent to the original tar concentration of ≤15 mg/ml), whereas some part of the cytotoxic activity is leaked with more than 8 cigarettes (equivalent to the original tar concentration of ≥20 mg/ml).

To confirm the leakage of the cytotoxic activity, another reservoir containing 15 ml of PBS was incorporated downstream of the first reservoir, and the cytotoxic activity of the PBS in the second reservoir was analyzed using MTS reduction assay. As shown in Fig. 4B, the PBS in the second reservoir showed no cytotoxic activity, when four cigarettes of Hi-Lite brand (equivalent to the original tar concentration of 10 mg/ml) were used for cCSE preparation, but it showed significant cytotoxic activity, when 14 cigarettes (equivalent to the original tar concentration of 35 mg/ml) were used, demonstrating that some part of cytotoxic activity has actually leaked.

These findings taken together show that as long as ≤6 cigarettes of Hi-Lite brand (equivalent to the original tar concentration of ≤15 mg/ml) are used for preparation of the original cCSE, nearly 100% of the cytotoxic activity is extracted into the PBS, but that with ≥8 cigarettes of Hi-Lite brand (equivalent to the original tar concentration of ≥20 mg/ml), part of cytotoxic activity leaks probably because of saturation of PBS. This means that when cCSEs are prepared at the original tar concentrations of ≤15 mg/ml, the tar concentrations are linearly related with the cytotoxic potency of cCSEs, and hence, that the tar concentrations can be used as a universal measure of cytotoxic potency of cCSE.

To exclude the possibility that the cytotoxicity is caused by a pH change in culture medium (DMEM) following addition of cCSE, we measured the pH values of the medium containing varying concentrations of cCSE. The pH values of DMEM containing cCSE at final tar concentrations of 0, 0.5 and 1.0 mg/ml were 7.48±0.02, 7.45±0.03 and 7.46±0.04, respectively (n = 3 for each group), which were not significantly different from each other. These results suggest that addition of cCSE to culture medium at least up to the virtual tar concentration of 1.0 mg/ml had no effect on the pH of culture medium.

### Effects of cigarette brands on the cytotoxicity of cCSE

To clarify whether the potency of cytotoxic activities of cCSE varies depending on cigarette brands, we examined the cytotoxicity of 8 representative cigarette brands (5 brands from JT, Japan, 3 brands from other countries) with different tar contents (Table 2). To prepare the original cCSEs at comparable tar concentrations from various brands of cigarettes, we first determined the dry weight of tar per cigarette which was trapped on the Cambridge filter after combustion of one cigarette by continuous smoking protocol (Table 2). The dry weight of tar per cigarette varied from 18.8 mg to 53.1 mg, which were two to three times larger than the tar content provided by the tobacco company: the difference is mainly due to that the tar content is determined by puff smoking according to the regulation of ISO3308, which discards cigarette smoke during the time interval except puff.

**Table 2:** Tar content per cigarette of various brands, the dry weight of tar trapped on the Cambridge filter after combustion of one cigarette and the EC_50_ values of cCSEs for inhibition of MTS reduction activity.

Cigarette brand	Nicotine content per cigarette (mg)^a^	Tar content per cigarette (mg)^a^	Dry tar weight per cigarette (mg)^b^	EC_50_ (mg/ml) for MTS reduction activity^c^
Peace	2.3	28	53.1±2.6	0.521±0.025
Hi-Lite	1.4	17	35.5±1.8	0.463±0.014
Seven Stars	1.2	14	35.6±0.4	0.502±0.023
Mevius	0.8	10	25.9±3.1	0.520±0.019
Mevius Super Light	0.5	6	18.8±2.5	0.534±0.006
Marlboro	1.0	12	26.1±1.1	0.496±0.028
Lucky Strike	1.0	11	22.7±2.4	0.495±0.029
Kent 9 mg	0.8	9	26.4±2.4	0.521±0.025

a Nicotine and tar content per cigarette is the value reported by its manufacturer and determined by puff smoking based on ISO regulation.

b For determination of dry tar weight per cigarette, smoke of one cigarette from either brand was continuously sucked through the Cambridge filter, and the increase in the dry weight of the filter was determined (represented as means ± SD of three experiments).

c For determination of the EC_50_ values, cCSEs at the original tar concentration of 10 mg/ml were prepared from cigarettes of various brands by continuous smoking, and subjected to MTS reduction assay in C6 glioma cells for construction of concentration-response curves from which the EC_50_ values (means ± SE of three experiments, each in triplicate) were determined.

**Figure 5:**
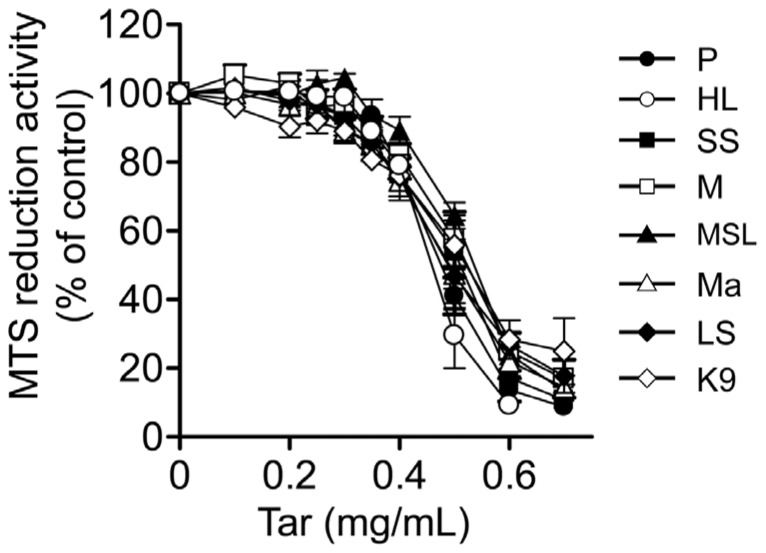
Relationship between cytotoxic activities of gas phase extracts of cigarette smoke and cigarette brand. The gas phase extracts of cigarette smoke (designated cCSE) at the original tar concentration of 10 mg/ml were prepared from cigarettes of various brands by continuous smoking protocol as described in the legend for Fig. 1. The cCSEs were subjected to MTS reduction assay for evaluation of their cytotoxic activities, as described in Fig. 3. MTS reduction activity in the absence of the gas phase extract was represented as 100%. Values represent means ± SE of three experiments, each in triplicate. P, Peace (JT, Japan; 28 mg tar, 2.3 mg nicotine), HL, Hi-Lite (JT, Japan; 17 mg tar, 1.4 mg nicotine), SS, Seven Stars (JT, Japan; 14 mg tar, 1.2 mg nicotine), M, Mevius (JT, Japan; 10 mg tar, 0.8 mg nicotine), MSL, Mevius Super Light (JT, Japan; 6 mg tar, 0.5 mg nicotine), Ma, Marlboro (Phillip Morris, USA; 12 mg tar, 1.0 mg nicotine), LS, Lucky Strike (British American Tobacco, UK; 11 mg tar, 0.9 mg nicotine), K9, Kent 9 mg (British American Tobacco, UK; 9 mg tar, 0.8 mg nicotine).

From these cigarettes, we prepared cCSEs, whose original tar concentration was 10 mg/mL (equivalent to the tar concentration of cCSE prepared from four cigarettes of Hi-Lite brand). The concentration-response relationships for inhibition of MTS reduction activity were not significantly different among the cCSEs prepared from different cigarette brands, as demonstrated by comparable values for the EC_50_ and maximal inhibition (Fig. 5 and Table 2). These results demonstrate that the cytotoxic activities of cCSEs depend on the tar concentration but not on either cigarette brands or nominal tar contents of cigarettes. Furthermore, the present results suggest that although cigarettes are highly engineered products containing differing tobacco leave composition and chemical additives [28], [29], those factors have little effect on the cytotoxic activities from the toxicological viewpoint.

### Comparison of cytotoxic potency of cCSE and pCSE

We compared the cytotoxic potency of two types of CSE, i.e. cCSE and pCSE, which were prepared by continuous or puff smoking protocols, respectively. For both CSEs, the original solutions were prepared at the virtual tar concentration of 10 mg/ml and their cytotoxic activities were examined using MTS reduction assay and DNA fragmentation assay for cell death, and LDH leakage assay and PI uptake assay for plasma membrane damage. For preparation of 15 mL of cCSE at that tar concentration, four cigarettes of Hi-Lite brand were required, while nine cigarettes were required for preparation of the same amount of pCSE.

**Figure 6:**
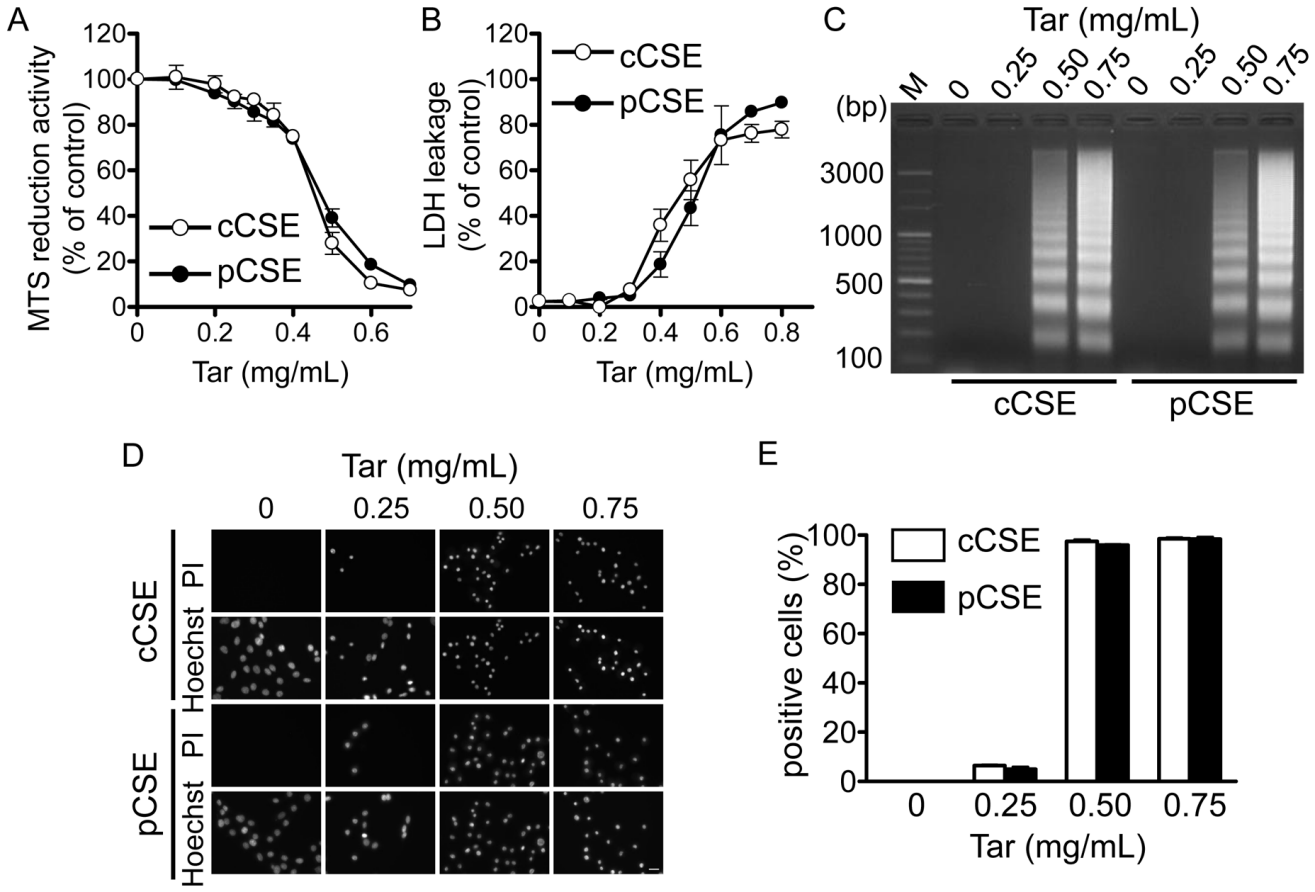
Cytotoxic activities of gas phase extracts of cigarette smoke and smoking methods. The gas phase extracts of cigarette smoke were prepared from Hi-Lite brand cigarettes by either continuous smoking protocol (cCSE) as described in the legend for Fig. 1 or puff smoking (pCSE) as described in Materials and Methods. The original gas phase extracts at the virtual tar concentration of 10 mg/ml PBS were prepared, and they were subjected to MTS reduction assay (A), LDH leakage assay (B), DNA fragmentation assay (C) and PI uptake assay (D, E) in cultured C6 glioma cells for evaluation of their cytotoxic activities. In MTS reduction assay, substrate reduction activity was represented as a percentage of the value in the absence of the gas phase extract; in LDH leakage assay, LDH activity leaked into culture medium was represented as a percentage of total activity in the medium of cells lysed by 0.2% Triton X-100; in PI uptake assay, the number of the cells positive for PI uptake was represented as a percentage of total number of cells identified by Hoechst 33342 for nuclear staining. Values in panels A, B and E represent means ± SE of three experiments, each in triplicate. The results in panels C and D are representative of three separate experiments.

cCSE and pCSE showed similar concentration-response relationships for inhibition of MTS reduction activity (Fig. 6A), induction of LDH leakage (Fig. 6B), induction of DNA fragmentation (Fig. 6C) and induction of PI uptake (Figs. 6D and 6E): there was no significant difference between cCSE and pCSE regarding the EC_50_ values for inhibition of MTS reduction activity (0.454±0.004 mg/ml vs 0.469±0.009 mg/ml, respectively) and the EC_50_ values for induction of LDH leakage (0.465±0.035 mg/ml and 0.524±0.025 mg/ml). These results indicate that the cytotoxic potency and property of the gas phase extracts do not depend on smoking protocol.

### Comparison of pharmacological properties of cCSE and pCSE

**Figure 7:**
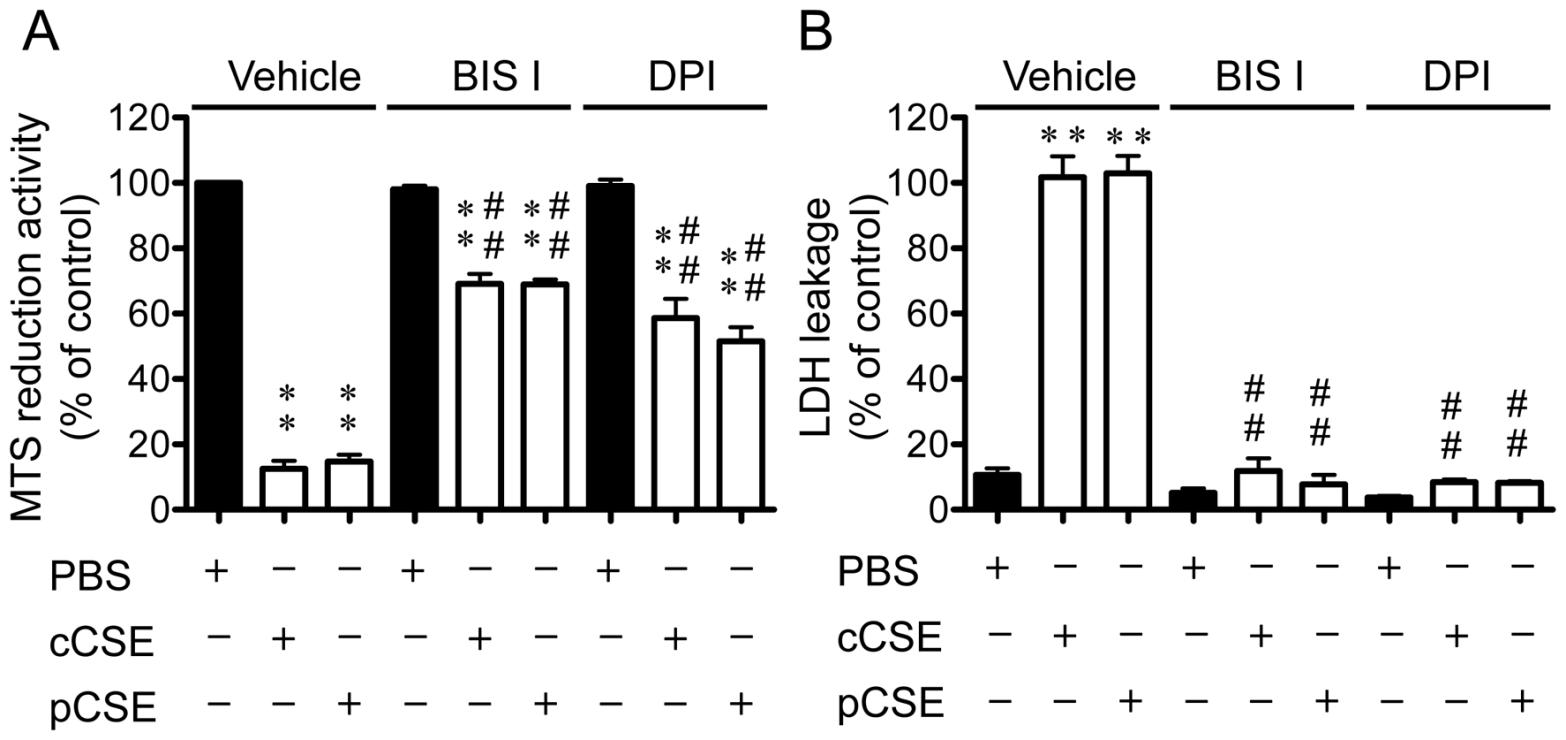
Pharmacological properties of cytotoxic activities of two types of gas phase extracts of cigarette smoke. The gas phase extracts of cigarette smoke at the virtual tar concentration of 10 mg/ml PBS were prepared from Hi-Lite brand cigarettes by either continuous (cCSE) or puff smoking protocol (pCSE), and they were subjected to MTS reduction assay (A) and LDH leakage assay (B). For determination of the effects of inhibitors of protein kinase C or NADPH oxidase, 5 µM BIS I or 1 µM DPI was added to the culture medium of C6 glioma cells, respectively, 30 min before the start of 4-h incubation with cCSE or pCSE. In panel A, MTS reduction activity was represented as a percentage of the control value in the absence of CSEs (PBS) within the vehicle-treated group. In panel B, LDH activity leaked into culture medium was represented as a percentage of total activity in the medium of cells lysed by 0.2% Triton X-100. Values represent means ± SE of three experiments, each in triplicate. **P<0.01 vs PBS-treated cells within either of three groups (Vehicle-, BIS I- and DPI-treated groups); ^##^P<0.01 vs cCSE- or pCSE-treated cells within the vehicle-treated group.

In our recent paper [19], [20], we have reported that cCSE induces the plasma membrane damage and cell death in cultured C6 glioma cells, and that total of the plasma membrane damage and part of cell death are induced by ROS which are produced by PKC-dependent activation of NADPH oxidase (NOX), based on the sensitivities to a PKC inhibitor (BIS I) and a NOX inhibitor (DPI). To get insights into the molecular mechanism of action of both types of CSEs, we compared the effects of BIS I and DPI on the cCSE- and pCSE-induced changes in MTS reduction activity (Fig. 7A) and LDH leakage (Fig. 7B).

Following exposure for 4 h to cCSE (final tar concentration, 0.6 mg/ml), MTS reduction activity was decreased to about 10% of the control value without the exposure, and the cCSE-induced decrease in MTS reduction activity was partially recovered (to approximately 70% of the control value) by pretreatment with the maximally effective concentration of BIS I or DPI (Fig. 7A), as reported recently [19], [20]. Exposure for 4 h to pCSE (final tar concentration, 0.6 mg/ml) also induced a decrease in MTS reduction activity to the same extent as exposure to cCSE, and the decrease was partially recovered by pretreatment with BIS I or DPI to the extent comparable to that induced by cCSE (Fig. 7A).

LDH leakage was increased to about 100% following exposure for 4 h to cCSE (final tar concentration, 0.6 mg/ml), and the increase was almost totally abrogated by pretreatment with the maximally effective concentration of BIS I or DPI (Fig. 7B), as reported [19], [20]. Exposure for 4 h to pCSE (final tar concentration, 0.6 mg/ml) also induced an increase in LDH leakage to the same extent as exposure to cCSE, and the increase induced by pCSE was suppressed by pretreatment with BIS I or DPI to the extent comparable to that induced by cCSE (Fig. 7B). These results taken together indicate that the action mechanisms of two types of CSEs (cCSE and pCSE) for cytotoxicity such as cell death and plasma membrane damage are similar from the pharmacological viewpoint.

### Comparison of the concentrations of carbonyl compounds in cCSE and pCSE

In our recent paper [23], we fractionated cCSE into nine fractions with HPLC, found two HPLC fractions to possess cytotoxic activities with functional assays in cultured cells and in those active HPLC fractions, identified ACR, MVK and CPO as stable cytotoxic factors responsible for the cytotoxic activities of cCSE. In addition, in the active HPLC fractions, we identified other carbonyl compounds such as acetone and propionaldehyde which do not possess cytotoxic activities. Therefore, we compared the concentrations of these carbonyl compounds in cCSE with those in pCSE (Table 3). For determination of the concentrations of these carbonyl compounds, both CSEs were first fractionated by reversed-phase HPLC and each fraction was analyzed for cytotoxicity. The fractions showing cytotoxic activities detected by PI uptake assay were subjected to GC/MS after derivatization with a carbonyl reagent PFBOA. As shown in Table 3, there was no significant difference between both types of CSE regarding the concentrations of cytotoxic carbonyls such as ACR and MVK and of noncytotoxic carbonyls such as acetone and propionaldehyde. These results demonstrate that the chemical composition of cCSE and pCSE is equivalent in terms of the concentrations of the major carbonyl compounds.

**Table 3:** Comparison of concentrations of carbonyl compounds in cCSE and pCSE.

	cCSE^a^	pCSE^a^
Acetone (µM)	287.9±29.2	326.1±20.3
Acrolein (µM)	36.7±1.3	41.7±2.8
Propionaldehyde (µM)	24.4±3.5	28.7±6.9
Methyl vinyl ketone (µM)	17.5±3.0	13.8±0.5

a The cCSE and pCSE at the original tar concentration of 10 mg/ml were prepared from Hi-Lite brand cigarettes by either continuous (cCSE) or puff smoking protocol (pCSE).

cCSE and pCSE were fractionated by HPLC and each fraction was analyzed for cytotoxic activities using PI uptake assay. The positive fractions were analyzed by GC/MS after derivatization with a carbonyl reagent PFBOA. Values represent means ± SD of three experiments.

### The sensitivity of various cell lines to cCSE

**Figure 8:**
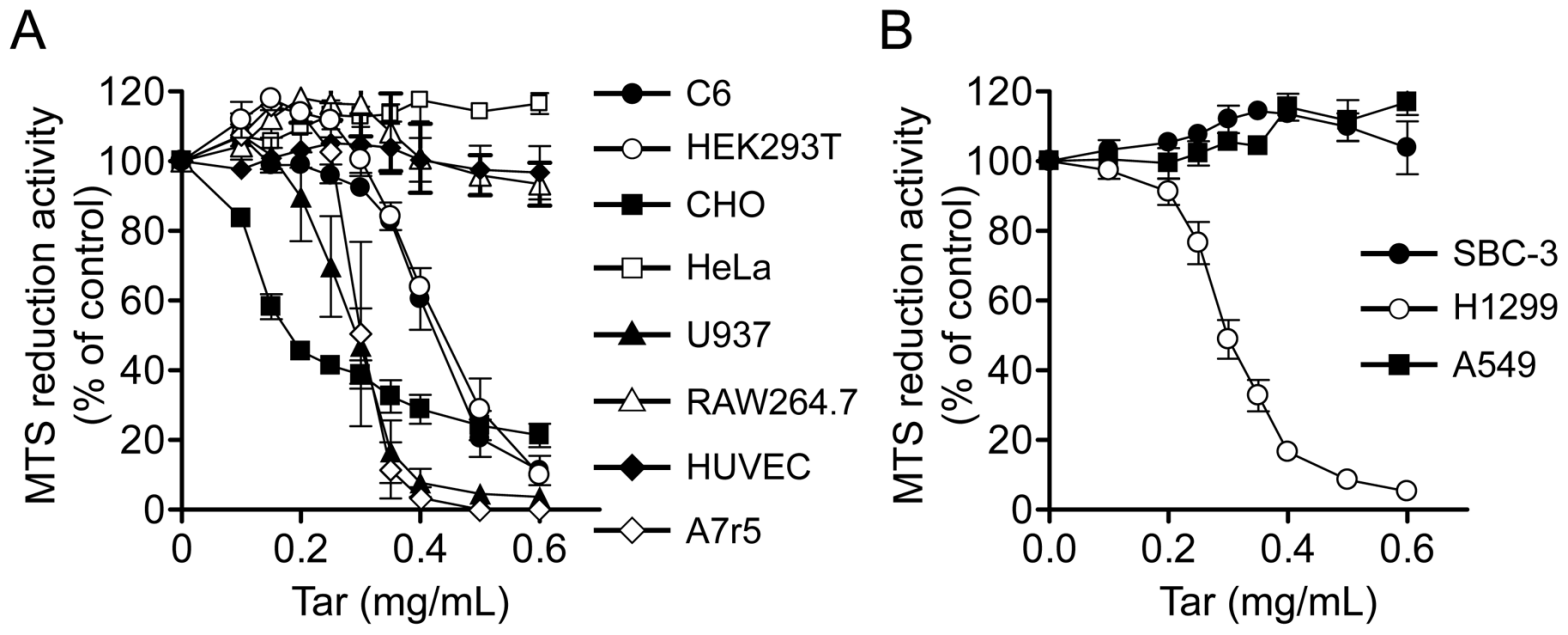
Sensitivities of various cultured cells to the gas phase extracts of cigarette smoke. The gas phase extracts of cigarette smoke (cCSE) at the virtual tar concentration of 10 mg/ml were prepared from Hi-Lite brand cigarettes by continuous smoking protocol, and they were subjected to MTS reduction assay using various cultured cells. MTS reduction activity was represented as a percentage of the control value in the absence of cCSE. Values represent means ± SE of three experiments, each in triplicate. (A) C6, rat glioma cells; HEK293T, human embryonic kidney cells; CHO, Chinese hamster ovary cells; HeLa, human cervical carcinoma cells; U937, human monocytes; RAW264.7, mouse macrophages; HUVEC, immortalized human umbilical vein endothelial cells; A7r5, rat aorta smooth muscle cells. (B) SBC-3, human lung small cell carcinoma; H1299, human lung squamous cell carcinoma; A549, human lung adenocarcinoma.

Finally, we examined the sensitivity to cCSE of various cell lines which are widely used, using MTS reduction assay (Fig. 8). Among these cell lines, CHO cells were the most sensitive to cCSE only in the low tar concentration range (up to 0.2 mg/ml), but they became relatively resistant in the higher tar concentration range. U937 human monocytes, A7r5 rat aorta smooth muscle cells and SBC-3 cells were the second sensitive: the EC_50_ values of cCSE for inhibition of MTS reduction activity were 0.285±0.021 mg/ml, 0.299±0.018 mg/ml and 0.300±0.010 mg/ml (represented by the tar concentration), respectively. C6 rat glioma cells and HEK293T cells were the third in the sensitivity, with the EC_50_ values of 0.423±0.019 mg/ml and 0.446±0.015 mg/ml, respectively. In contrast, HeLa cells, RAW264.7 mouse macrophages, HUVEC, H1299 cells and A549 cells were resistant to CSE up to the tar concentration of 0.6 mg/ml.

## Discussion

The gas phase of cigarette smoke is considered to be important from the viewpoint of human health, because it is the gas phase but not the tar phase that can pass through the alveolar epithelium of the lung to enter the circulation, exerting cytotoxic effects in tissues remote from the lung [14], [15], [30]. However, no standard method for preparation of the gas phase extracts of cigarette smoke has so far been established, and hence, researchers have performed experiments using the gas phase extracts prepared by their own methods. Because of the potential variability in the composition and concentration of those extracts, the comparison of the data from different laboratories has been difficult. In the present study, we have standardized a simple and rapid method for preparation of the gas phase extract of cigarette smoke based on continuous smoking protocol (referred to as cCSE).

The protocol for preparation of cCSE established in the present study is as follows. 1) Cigarette smoke of any brand is continuously sucked through a Cambridge filter with a constant flow rate of 1.050 l/min. 2) The smoke is subsequently bubbled through a glass ball filter of normal pore size into 15 mL of PBS kept at 25°C. 3) The dry weight of the tar phase trapped on the Cambridge filter is determined after drying at 25°C for 12 h, 4) The concentration of cCSE is expressed in terms of the virtual tar concentration which is calculated on the assumption that the tar trapped on the Cambridge filter is dissolved in the PBS used for cCSE preparation, 5) Combustion of cigarette can be repeated as long as the dry weight of tar trapped on the Cambridge filter is ≤225 mg.

The most important finding is that the concentration of cCSE expressed in terms of the virtual tar concentration can be used as a reference value to normalize the cytotoxic activities of cCSE, irrespective of the number of combusted cigarettes, cigarette brands and smoking protocols (continuous smoking vs puff smoking), as long as the tar concentrations in the original cCSEs are ≤15 mg/ml of PBS: over this concentration range, part of the cytotoxic activity in the smoke escapes without being extracted into the PBS, causing a lower cytotoxic potency than expected from the tar concentration.

Amongst cigarette brands, the ratio of tar to other gas phase components might vary. Indeed, the ratio of tar to nicotine varies among brands (Table 2). Given this, a given virtual tar concentration may expose cells to differing levels of specific gas phase components despite standardizing by the virtual tar concentration. However, as shown in Figure 5, the concentration-response curves for the cytotoxic activities of the cCSEs prepared from different cigarette brands are not significantly different from each other, when the concentrations of cCSEs are normalized in terms of the virtual tar concentration. This result strongly indicates that the concentrations of cytotoxic compounds in the CSEs are actually normalized by the virtual tar concentration, and hence that the ratio of cytotoxic compounds to tar is independent of the ratio of nicotine to tar.

Another important finding is that the toxicological properties of cCSE are equivalent to those of pCSE, in terms of potency of cytotoxicity, pharmacology of the cytotoxicity and the concentrations of major cytotoxic compounds such as ACR and MVK. Although there are no experimental data, it has so far been believed that the chemical composition and hence the property of the cytotoxicity of the smoke generated by continuous and puff smoking protocols might be different, mainly based on the consideration that the combustion temperature of cigarette might be different between the two smoking protocols, leading to generation of different spectrum of chemicals [31], [32].

The standard method for cCSE preparation established in the present study makes possible the comparison of the experimental data on cCSE from various laboratories, and is expected to accelerate the research on toxicity of smoking and pathophysiology of smoking-related diseases, leading to development of methods for prevention and treatment of smoking-related diseases. However, because cytotoxicity is only one measure of the effects of cigarette smoke, it is noted that other measures such as protease/cytokine expression or mucin production [33], [34] might vary among brands despite the virtual tar concentration being controlled.

In the present study, we have proposed a standardized method for preparation of nicotine- and tar-free CSE, i.e. the gas phase extract of cigarette smoke. The other phase (tar phase) containing tar and nicotine is reported to be also important for human health [35]–[37]. However, a standardized method for preparation of the tar phase extract which is simple and rapid is also absent. Therefore, as a next step, it is important to establish such standardized method and to accelerate the investigation on the cytotoxic effects of nicotine and tar and their molecular mechanisms of action.

In summary, we introduced the virtual tar concentration as a measure of cytotoxic potency of the gas phase extract of cigarette smoke, and established a simple and rapid method for standard preparation of the gas phase extract of cigarette smoke based on continuous smoking protocol. We also demonstrated that from the toxicological viewpoint, the gas phase extract prepared by the present method is equivalent to the extract prepared by a puff smoking machine.
